# Expected number of quantum channels in quantum networks

**DOI:** 10.1038/srep12128

**Published:** 2015-07-15

**Authors:** Xi Chen, He-Ming Wang, Dan-Tong Ji, Liang-Zhu Mu, Heng Fan

**Affiliations:** 1 School of Physics, Peking University, Beijing 100871, China; 2 Department of Physics, Tsinghua University, Beijing 100084, China; 3 Beijing National Laboratory for Condensed Matter Physics, Institute of Physics, Chinese Academy of Sciences, Beijing 100190, China; 4 State Key Laboratory of Theoretical Physics, Institute of Theoretical Physics, Chinese Academy of Sciences, Beijing 100190, China

## Abstract

Quantum communication between nodes in quantum networks plays an important role in quantum information processing. Here, we proposed the use of the expected number of quantum channels as a measure of the efficiency of quantum communication for quantum networks. This measure quantified the amount of quantum information that can be teleported between nodes in a quantum network, which differs from classical case in that the quantum channels will be consumed if teleportation is performed. We further demonstrated that the expected number of quantum channels represents local correlations depicted by effective circles. Significantly, capacity of quantum communication of quantum networks quantified by ENQC is independent of distance for the communicating nodes, if the effective circles of communication nodes are not overlapped. The expected number of quantum channels can be enhanced through transformations of the lattice configurations of quantum networks via entanglement swapping. Our results can shed lights on the study of quantum communication in quantum networks.

Recently, quantum networks and their extension, the quantum internet, which may potentially be the next generation of the internet, have been attracting a great deal of interest^1^,^2^,^3^,^4^,^5^,^6^,^7^,^8^,^9^,^10^. Quantum networks consist of quantum nodes in which quantum information can be locally produced, stored and processed. Those nodes should be linked by both quantum channels and classical channels to allow quantum states to be teleported^11^ between different nodes. Quantum networks may have the capability to perform distributed quantum computation, simulation of quantum many-body systems^12^,^13^, quantum metrology^14^, or quantum cloud computations with unconditional security by means of quantum key distribution protocols^15^,^16^. These exciting features provide strong motivation to examine research related to quantum networks.

In quantum networks, quantum states can be sent directly between any two nodes by means of flying qubits such as photons. The prior-shared entangled states between different nodes, which can be considered as the available quantum channels, can facilitate quantum communication involving only local operations performed on each individual node with assistance of classical communication. Additionally, the fidelity of state transmission in teleportation can be increased when entanglement purification is applied^17^. The efficiency of such a quantum network for quantum communication should be well described and quantified. One pioneering work has examined the entanglement percolation in quantum networks^18^. It has been demonstrated that the problem of establishing maximally entangled states between nodes can be directly related to classical percolation in statistical mechanics. By applying appropriate local measurements to a portion of nodes via entanglement swapping^19^, the lattice configurations of quantum networks can be modified. The percolation threshold is consequently changed, allowing the entanglement percolation to switch from failure to success.

However, the entanglement percolation describes only whether a maximally entangled pair can be successfully constructed between two nodes, i.e., it only indicates ‘success’ or ‘failure’. If it succeeds, it is still not clear what amount of quantum information can be teleported. Unlike for classical networks, this is a crucial concern for quantum networks both in theory and in real-world quantum network constructions because once the pre-shared entanglement is consumed for telepotation, new entangled pairs should be distributed to construct new quantum channels. Thus, the expected number of quantum channels (ENQC), i.e., the expected number of qubits that can be teleported from certain sender to receiver, becomes important for quantum networks. A further consideration is whether the change in lattice configuration induced by entanglement swapping can increase the capacity for quantum communication. In this consideration the number of quantum channels can be a quantified indicator as well.

In this work, first, for regular quantum networks, we define ENQC as the expected number of maximally entangled pairs that can be formed between certain sender and receiver in an established quantum network, normalized by the node degree, i.e., the number of bonds connecting each node. It quantifies the number of qubits which can be teleported from the sender to the receiver. We then study two types of ENQC in regular networks and find that they depend on the local structure and the bonds’ singlet-conversion probability of the quantum network. In particular, ENQC is independent of the distance between the participating nodes when the nodes are separated by a distance greater than their effective radii. This means that, at long distance, the ENQC is not an exponentially decaying quantity, thus avoiding the primary hurdle for long-distance quantum communication. Moreover, we demonstrate that the ENQC can be increased through lattice transformations induced by quantum measurements on certain nodes. We presented discussions for other regular networks and arbitrary random networks in the Supplementary Information and ENQC is expected to be a universal quantity that can be applicable to quantum networks with various configurations.

## Results

### Expected number of quantum channels

**Figure 1:**
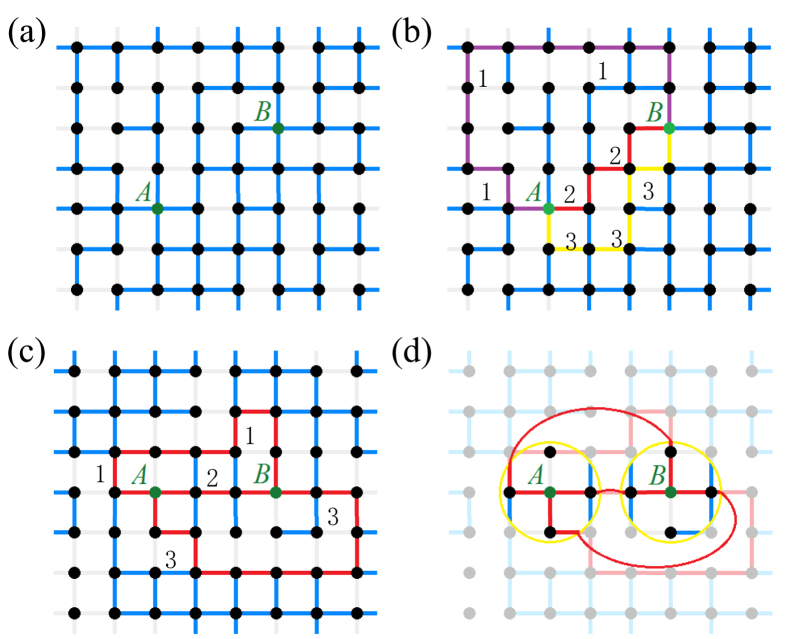
A portion of an infinite square network with *p* = 2/3 after singlet conversion. For convenience in these figures *A* and *B* are very close to each other. Actually the distance can be arbitrary. (**a**) The blue bonds represent states that have been successfully converted into maximally entangled states, whereas the gray bonds represent those that have failed. (**b**) The three independent quantum channels between *A* (Alice) and *B* (Bob), which are marked as green points, are highlighted in red, yellow and purple (also labeled as 1, 2 and 3). (**c**) The three quantum channels between Alice and Bob (green points), for another example, are highlighted in red (also labeled as 1, 2 and 3). (**d**) The attractive circles in the network of (**c**). Each circle contains 4 ‘internal bonds’. Outside these effective circles, the network can be regarded simply as an non-decaying ‘medium’. Thus, whether a quantum channel can be formed or not mostly depends on how many ‘internal bond’ is formed.

The main type of quantum networks studied in this article is regular quantum-networks, namely, periodic two-dimensional lattices. Here we took square lattice as an example, as depicted in Fig. 1(a). The nodes are located at lattice sites, and each bond connecting nearest-neighboring nodes corresponds to a pure entangled state shared by two nodes, 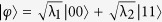, where real numbers λ_1_ + λ_2_ = 1 and λ_1_ ≥ λ_2_ is assumed.

Suppose Alice at node *A* in the network wished to transmit several qubits to Bob at node *B* following the standard protocol: First, Alice converted each entangled state into a maximally entangled one with optimal conversion probability^20^ *p* = 2(1 − λ_1_). The quantum network can thus be mapped to a graph in which each bond has an formation probability *p*, see Ref. 18 and Fig. 1(a). Second, if some of the maximally entangled states can form a path connecting *A* and *B*, then a maximally entangled state between *A* and *B* can be generated by repeated entanglement swapping on each intermediate node. Third, Alice can teleport a qubit to Bob. If the singlet conversion probability *p* for a certain network is larger than the percolation threshold of the network, an infinite component can be connected by maximally entangled states and thus quantum communication can be successful even between two sites with infinite distance^18^.

The quantification of efficiency of classical and quantum networks can be considerably different. In the classical network, once the information transmission channels, like cables, are constructed, information can be transmitted through these channels without damaging them. Thus, these channels can be used repeatedly and the efficiency of a classical network can be evaluated by, for example, its transmission rate, namely the information transmitted per unit of time. However, a quantum channel can be used only once. After teleporting a qubit through a quantum channel, the entanglement resource would be consumed and the bonds no longer carried any entanglement. For example, given a quantum network and two sites of it as the sender and the receiver, the number of qubits that the sender can transmit to the receiver is equal to the number of quantum channels existing between the sender and the receiver. Thus, the ENQC we proposed can be a good quantity to describe the average efficiency of quantum communication.

We defined the ENQC as the ratio of expected number of maximally entangled pairs that can be successfully formed between *A* and *B* to the cost of constructing a quantum network,


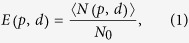


where *E* denotes ENQC, 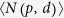 is the expected number of maximally entangled pairs connecting *A* and *B*, separated by a distance *d*, which is defined to be the linear distance between these two nodes. For instance, the distance of two neighboring nodes is defined as *d* = 1 and that of two diagonal-neighboring nodes is defined as 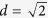. *N*_0_ is the degree of nodes of a lattice. For example *N*_0_ = 4 for square lattice and *N*_0_ = 6 for triangle lattice. *p* is the singlet conversion probability. We have 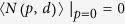 and 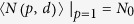. Suppose the total number of nodes *K* is fixed in a given quantum network, *N*_0_ represents the number of initial non-maximally entangled states distributed in the network, namely the cost of network construction *C* = *N*_0_*K*/2. Thus only *N*_0_ is presented in the definition in order to make ENQC to range from 0 to 1. ENQC increases with increasing *p* and a larger ENQC indicates higher efficiency of qubit transmission. An visual illustration is given in Fig. 1(b). For a certain network, ENQC is the function of *p* and *d*. But the parameters in the function varies with different network structures. Note that the ENQC is defined as an average quantity, which is either averaged over all nodes in a network, or averaged over a network ensemble.

Generally it can be difficult to determine ENQC analytically because ENQC involves not only the features of nodes and bonds, but also the feature of paths in the networks, namely, the network structure. However, as we can see below, the regular structure of lattices allows us to construct a rather simple model that can reasonably approximate ENQC.

### Attractive and Repulsive Radii

The explicit expression of ENQC can be determined both numerically and analytically. Here, we still considered a square lattice. We also assumed that, in our square lattice example, *p* > 0.6, which clearly exceeded the percolation threshold of a square lattice, *P*_*critical*_ = 0.5. When *p* is lower than the critical point, 0.5, the ENQC will experience an exponential decay with zero long-distance ENQC and when *p* exceeds 0.5 but not much, the quantum-state transmission efficiency can be reasonably assumed to be low and the effective approximation method presented. More complicated analysis may be required. Thus it is not of our interest here to discuss situations where *p* is near *P*_*critical*_.

**Figure 2:**
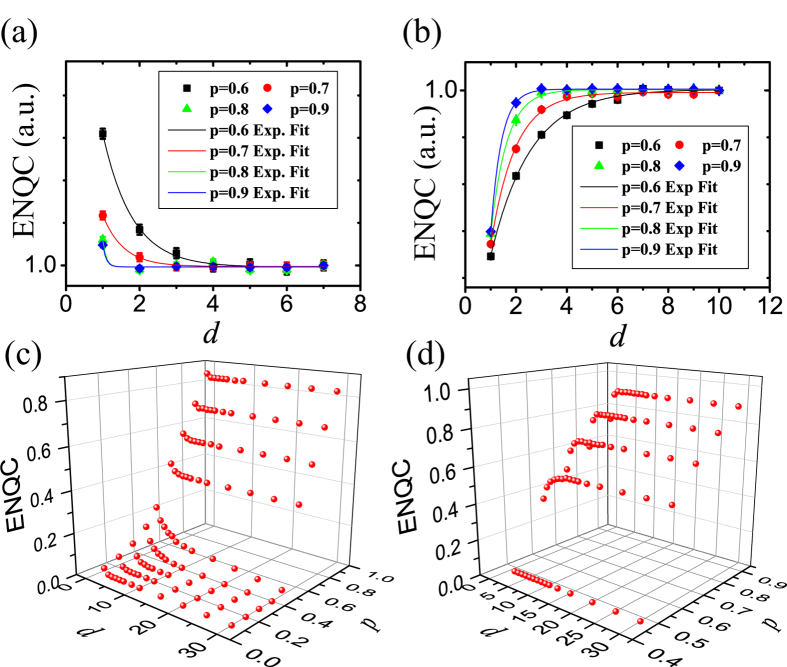
The behavior of ENQC showing the locality characteristic. (**a**) Monte Carlo estimations of ENQC between two nodes in a square lattice for various values of *p*. Because the attractive radii pertain to relative change in ENQC, we depict the relative ENQC, such that all ENQCs of *d* = 7 are normalized to 1. For the original images, see Supplementary Information for details. The corresponding *γ* values for *p* = 0.6, 0.7, 0.8, and 0.9 are 1.2, 1.7, 10.6, and 10.9, with *r*_*at*_ = 1.33, 1.09, and 1, respectively. A rather rapid convergence can be observed when *p* > 0.8. (**b**) Monte Carlo estimations of the ENQC from two nodes to infinity for various values of *p*. For convenience in demonstrating the repulsive radii, we again present the relative ENQC. All ENQCs for *d* = 10 are normalized to 1. For the original images, see Supplementary Information for details. The corresponding *γ* values for *p* = 0.6, 0.7, 0.8, and 0.9 are 0.64, 1.04, 1.59, and 2.37, with *r*_*rp*_ = 2.06, 1.46, 1.13, and 1, respectively. In our simulations the node number *N* ≈ 10^4^ and the error bars are presented in the figures. (**c**), (**d**) Three dimensional figures showing ENQC as a function of *d* and *p* under the same conditions of (**a**) and (**b**). In (**c**) simulation results of *p* = 0.1, 0.2, 0.3, 0.4, 0.48, 0.6, 0.7, 0.8, and 0.9 are presented. Clearly, *p* < *p*_*th*_ = 0.5 corresponds to *E*_0_ = 0. In (**d**) simulation results of *p* = 0.48, 0.6, 0.7, 0.8, and 0.9 are presented. In this protocol *p* < *p*_*th*_ gives *E*(*p*, *d*) ∫ 0.

Let Alice and Bob be located on two different lattice nodes *A* and *B* separated by a distance *d*, as defined in Sec. II. Alice would like to send several qubits to Bob via the network. We used Monte Carlo simulation to investigate ENQC. Basically our simulation included first generating networks with different singlet-conversion probabilities *p*, which correspond to successfully formed maximally entangled states. Second we counted the numbers of exclusive paths connecting two given nodes in repeated simulations. These numbers exactly represented the numbers of maximally entangled pairs that can be formed between *A* and *B*. The uncertainty of the results are approximately 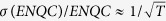, where *σ*(*ENQC*) is the uncertainty and *T* is the number of repeated simulations. The results (Fig. 2(a)) indicate that when *p* > 0.6, ENQC between *A* and *B* decreases and converges quickly to its asymptotic value as *d* increases. This rapid convergence suggests us to define the attractive radius of an effective circle. We then can fit the ENQC using an exponential function,


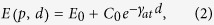


where *E*_0_, *C*_0_ and *γ*_*at*_ are parameters that depend on *p* and network structure. If *p* < *p*_*critcal*_, *E*_0_ = 0 which corresponds to exponential decaying of quantum communication. The attractive radius is introduced as an exponential parameter,


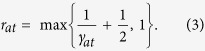


This radius is illustrated in Fig. 1(c,d). Because of the discreteness of nodes and the minimal distance is 1, we required *r*_*at*_ to have an lower bound 1, namely for the effective circle to contain at least the neighboring points. When *d* > 2*r*_*at*_, i.e., the two attractive circles do not overlap, the second term in Eq. (2) can be neglected and *E*_0_ asymptotically approaches *E*(*p*,*d*). Thus *E*_0_ would be the quantity of interested for long-distance communication. We presented an estimation of *E*_0_ at the end of this section and in Supplementary Information. Eq. (2) agrees well with numerical results, see Fig. 2(a). For *p* > 0.6, we have a rough estimation for the attractive radius, 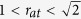. This means the effective circle of *A* or *B* includes only the nearest-neighboring nodes, respectively, on a square lattice.

The physical interpretation of *r*_*at*_ can be understood as follows. When the distance between *A* and *B* is larger than 2*r*_*at*_, the ENQC will be approximately independent of the distance, *ENQC* = *E*_0_, which is a fixed value for a certain lattice of certain singlet conversion probability *p*. It seems the two nodes *A* and *B* can be connected by first connecting from their sites to the borders of their effective circles and then connecting the two circles through the non-decaying medium. Thus, the effective radius can be regarded as a ‘correlation length’. Our numerical results are consistent with the conclusion of quantum percolation theory in that if quantum percolation succeeds (fails), we have corresponding nonzero (zero) *E*_0_ . Thus avoids the usual difficulty of exponentially decaying probability for long-distance quantum communication. The ENQC and attractive radius further quantifies the number of quantum channels that are available according to different positions of sender and receiver in a quantum network. When *A* and *B* are located closely with their attractive radii overlapped, it may be possible to construct more quantum channels connecting the two nodes. Therefore, the attraction will be beneficial, which motivates the name ‘attractive radius’.

Moreover, we considered another scheme similarly, in which Alice and Bob would like to send qubits to many receivers located very far away in the network. We proposed this because in real-world quantum networks, information may be send collectively and cooperatively as what we discussed in this proposal. We expected that when *A* and *B* located closely, their ability of sending out qubits would be influenced because quantum channels may run into each other thus decreased the number of available quantum channels. Thus, we similarly defined a repulsive radius, *r*_*rp*_. *A* and *B* can be regarded as independent if the distance *d* > 2*r*_*rp*_. Monte Carlo simulations indicate that when *p* > 0.6, this type of ENQC can also be described well by an exponential function (Fig. 2(b),


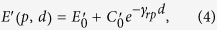


where *d* is the distance between *A* and *B*, *r*_*rp*_ = max{1/*γ*_*rp*_ + 1/2,1} and 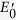 and 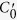 are parameters similar to those used in the previous expressions of the attractive radius. Different from *C*_0_ in Eq. (2), *C*_0_ is negative here. Because the receiver is located at infinite distance, *p* < *p*_*th*_ corresponds to 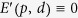. For *P* > 0.6, we have the following rough estimation: 1 < *r*_*rp*_ < 2. This means if the distance between *A* and *B* is larger than 2*r*_*rep*_ ≈ 4, they can be considered to be independent with regard to qubit transmission. As we can see, the approach of effective radius can be applicable in maximizing qubit transmission efficiency of different protocols in quantum networks. In the rest parts of this work, we focused on the attractive radius, which describes the exponential decay of ENQC to a nonzero value. In Fig. 2(c,d) we have presented ENQC as function of *d* and *p* simultaneously in three-dimensional graphs. Not surprisingly, the ENQC remains unchanged with large distance. The effective radius can be rather small while *p* is not very close to the percolation threshold *p*_*th*_. These results might further support our result Eq. (2) and Eq. (4), and is in accordance with percolation theory, for *p* > *p*_*th*_ corresponds to a non-zero *E*_0_ and *p* < *p*_*th*_ corresponds to a zero *E*_0_.

**Figure 3:**
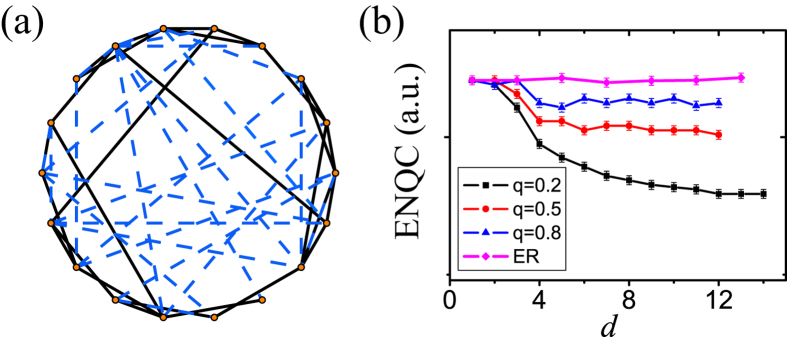
Effective attractive radius of small-world networks and Erdös-Rényi networks. (**a**). Figure of small-world and Erdös-Rényi networks. Black solid lines are for small-world network with *K* = 4 defining the number of initial bonds, *p* = 0.8, *q* = 0.2 being the switching probability quantifying the randomness. The blue dashed lines are for Erdös-Rényi network which is complete random corresponding to *q* = 1. (**b**). Monte Carlo estimation of ENQC of networks with different switching probability *q*. For Erdös-Rényi network with *q* = 1, ENQC has no decaying. When the randomness is reduced for decreasing *q*, ENQC gradually demonstrate decay to exponential-like decay suggesting that the Eq. (2) and effective radii depend on the short-distance structure of the regular lattices. In our simulations the node number *N* ≈ 10^3^ and the error bars are presented in (b).

The behavior of ENQC that they converge exponentially might root in the short-distance structure of the studied regular lattices. As a supporting example, we investigated the small-world network^21^, which is a random network whose “randomness” can be controlled by the rewiring probability *q*. Two kinds of small-world networks with extreme *q* are regular lattices (regular network) and the Erdös-Rényi (ER)^22^ network, namely the completely random graph. We found that the effective radii phenomena disappears gradually when the “randomness” of the network increases. That is, the behaviors of ENQC may range from an exponential decay to non-decay at all. The model and numerical results are presented in Fig. 3 and more details are presented in the Supplementary Information.

Because one of the main aim of quantum network is the communication between distant nodes, the long-distance ENQC, *E*_0_, would be more important for communication than other parameters. When the distance between the two nodes *A* and *B* is 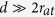, we have ENQC = *E*_0_ as shown in (2). This distance-independent quantity is determined by network structure and singlet-conversion probability *p*, and can be approximated analytically for *p* > 0.6 when it is far beyond the percolation threshold, as already pointed out previously. Starting from node *A*, the probability that the quantum channel does not meet a dead end in the ‘medium’ can be written as (1 − (1 − *p*)^3^)^*α*^, where *α* is like an index of the medium depending only on structure of network and can be fitted by numerical data. Thus, *E*_0_ depends on *p* and network structure. An estimated ENQC can be written as follows,


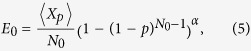


where 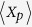 is the expected value of the random number of pairs *X*_*p*_, constructed between *A* and *B*.


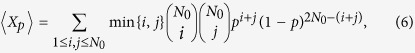


**Figure 4:**
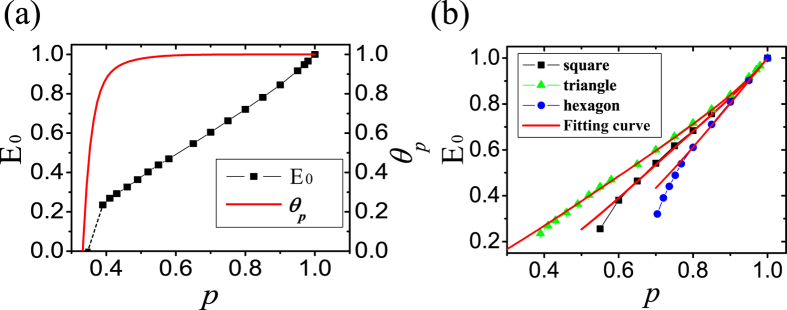
Asymptotic value of ENQC. (**a**) Comparison between ENQC (*E*_0_) and *θ*_*p*_ of triangle lattice(the proportion of nodes in the largest connected nodes)^23^,^26^. We may observe that *θ*_*p*_ corresponding to entanglement percolation is a jump function demonstrating only ‘success’ or ‘failure’, while the behavior of the ENQC (*E*_0_) is different, it in general increases monotonically with *p* and approaches 1 finally when *p* takes the limit of 1. **(b**) Lines with different symbols represent Monte Carlo estimation of ENQC (*E*_0_) of square, triangle and hexagon lattices. Red lines are fitted curves according to Eq. (5), with corresponding lattice indices *α*_*square*_ ≈ 2.6, *α*_*triangle*_ ≈ 1.0, *α*_*hexagon*_ ≈ 2.8. In our simulations the node number *N* ≈ 10^4^ and the error bars are presented in the figures.

*N*_0_ = 4 is the degree of nodes for square lattice. Detailed deduction is presented in the Methods. This formula agrees well with the simulation data, see Fig. 4(b). Note that Fig. 4(a) shows difference between entanglement percolation and ENQC. We expect that our method can be applicable to other regular networks.

### Lattice Transformation

Lattice transformations induced by entanglement swapping at certain nodes can possibly enhance entanglement percolation properties^18^,^23^ including transforming a network with *p* < *p*_*critical*_ to a network with *p* > *p*_*critical*_. In this section, we focused on the behavior of the long-distance ENQC, *E*_0_, of networks upon various transformations and study whether the lattice transformations can increase the communication efficiency. The ENQC of the network after transformation can be written as follows, 
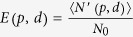
, where 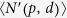 refers to the expected number of maximally entangled pairs connecting *A* and *B* in the new network and *N*_0_ is the corresponding quantity in the old network with *p* = 1 because the cost of the network is determined by previous network. If two or more bonds are present connecting two neighboring nodes of a network, each bond should be counted separately. Thus the comparison of the magnitude of the *E*_0_ before and after lattice transformation can be an evaluation of whether a proposal of lattice transformation is beneficial. If *E*_0_ is enhanced after lattice transformation, the transformation would be beneficial because more information can be transmitted in this network after transformation.

Using Monte Carlo methods, we observed two typical situations in which lattice transformations may be beneficial. In our simulations the node number *N* ≈ 10^4^ and the distance we chose are all greater than 20 to guarantee that we are simulating the long-distance ENQC, *E*_0_. Suppose the entanglement percolation thresholds is different for the lattices before and after transformation, if we draw the *E*_0_–*p* curves of the network before and after lattice transformation in one figure, they may exhibit two types of behaviors, **(i)** non-intersecting or **(ii)** intersecting. For the **(i)** non-intersecting case, lattice transformation can enhance the efficiency for all *p* above the percolation threshold. By contrast, for the **(ii)** intersecting case, the lattice transformation induced by entanglement swapping is beneficial in certain regions but not in others, i.e., the *E*_0_ may decrease after lattice transformation. In the following, we considered two lattice transformations as examples. A third case is presented in Supplementary Information.

**Figure 5:**
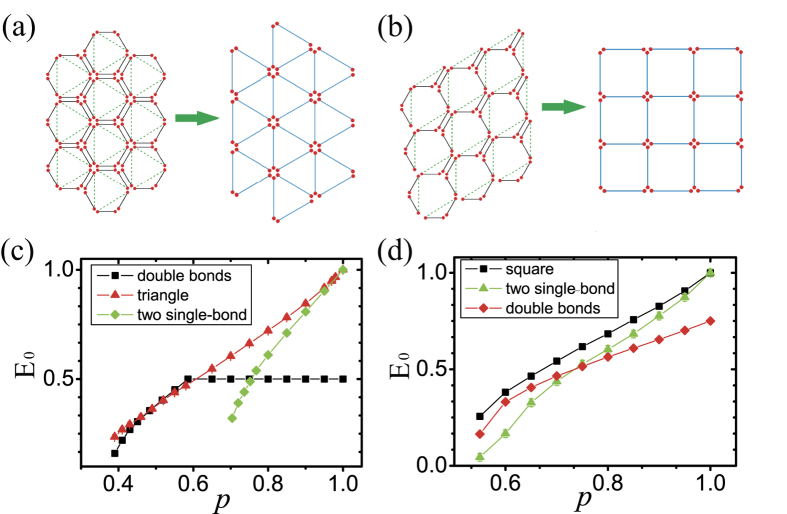
Lattice transformation. (**a**) The transformation of a double-bond hexagon lattice into a triangle lattice via entanglement swapping. Dashed lines represent outcomes of the entanglement swapping. (**b**) The transformation of a hexagon lattice with 1/3 double bond into a square lattice via entanglement swapping. Dashed lines represent outcomes of the entanglement swapping. (**c**) Comparison among the ENQC (*E*_0_) of a double-bond hexagon lattice (black dotted line), a single-bond triangle lattice (red dotted line) and two copies of a single-bond hexagon lattice (green dotted line). We can see that for approximately 0.5 ≤ *p* ≤ 0.6, the lattice transformation is not beneficial. In the limit *p* → 1, the triangle lattice and the two copies of single-bond yield almost the same ENQC (*E*_0_). (**d**) Comparison among the ENQC (*E*_0_) of a hexagon lattice with 1/3 double bonds (red dotted line), a square lattice (black dotted line) and a hexagon lattice in which double bonds are treated as two single bonds (green dotted line). We can see that after lattice transformation, not only is the percolation threshold decreased but the ENQC (*E*_0_) of the square lattice for all *p* is also greater than those two different treatments of the double bonds. In our simulations the node number *N* ≈ 10^4^ and the error bars are presented in the figures.

As presented in Fig. 5(a), a double-bond hexagon lattice with a value of *p* that is slightly less than its percolation threshold *p*_*c–hex*_ can be transformed into a single-bond triangle lattice with a value of *p*′ that is greater than its percolation threshold *p*_*c–tri*_ via entanglement swapping^18^,^23^. Our approach by estimating *E*_0_ reveals that there is an intersection between the two *E*_0_ curves. The *p* value corresponding to the intersection can be estimated by applying Eq. (5) to triangle and hexagon lattices: 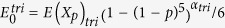 and 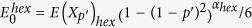, respectively. We can substitute 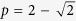 and *p*′ = 2(1 − (1 − *p*/2)^2^) into these equations. It is apparent that 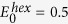, whereas when we apply Eq. (5) to the triangle lattice, we find that 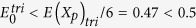, which indicates an intersection in the curves of *E*_0_ (see Fig. 5(c).

Monte Carlo estimations confirmed the existence of this intersection. Our approach provides an explicit evaluation of this lattice transformation: For *p* ≤ *p*_*intersect*_, lattice transformation is beneficial in achieving not only a lower critical point for the entanglement percolation but also a higher efficiency. However, for *p* ≥ *p*_*intersect*_, the lattice transformation will decrease the quantum communication efficiency.

Another possible treatment of double-bonds involves converting two single-bonds of a double-bond to maximally entangled pairs with probability *p*. This strategy can be applied for around *p* > 0.76 according to Fig. 5(c).

As another example, we consider a hexagon lattice with 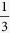 double-bonds, which can be transformed into a single-bond square lattice (Fig. 5(b). By investigating the former with *p* = 0.5, we can readily conclude that for both treatment of the double-bonds, the percolation threshold of the square lattice is lower than that of a hexagon lattice with 1/3 double-bonds (Fig. 5(d). Monte Carlo estimations show that the *E*_0_ of square lattice is higher than the *E*_0_s corresponding to the two different treatments of the double-bonds. These results indicate we can benefit from lattice transformation over the entire range of *p* > 0.5.

In this work, while we introduced the ENQC and its corresponding effective radii in regular networks with pure initial entangled-states, these notions are expected to work in a wide variety of networks and the initial entangled-states can be mixed states. The discussion of arbitrary random networks will be presented in the Supplementary Information. Our results about the ENQC can be extended to mixed entangled-states in a similar way. The entanglement percolation of bipartite mixed states including Werner states was investigated in Ref. 24. In that scheme, we can also find similarly the singlet conversion probability *p* for the conversion from a mixed state to a maximally entangled state. Thus, the ENQC can be similarly investigated as presented this work. Our conclusions remain the same for various lattices. For lattice transformations, we should rely on the entanglement swapping of mixed states where the basic technique is known^25^. After this entanglement swapping, we can obtain a new probability *p*′ for the new bonds. Whether or not the enhancement of ENQC would happen depends on two probabilities *p*,*p*′ and the corresponding lattice configurations.

## Discussion

We demonstrated that the efficiency of quantum-network communication can be well described in terms of the ENQC. Based on percolation theory, our approach of ENQC aims at answering the question of the efficiency of different protocol of quantum network. We studied the properties of the ENQC with respect of network structure and defined effective radius to quantify the local correlation, i.e., the influence of a node on others. The ENQC quantifies the amount of quantum information capable of being transmitted by teleportation, which is a fundamental protocol of quantum communication for quantum networks. Notably, the quantum communication efficiency defined by ENQC can be enhanced via lattice transformations induced by entanglement swapping. Our approach of ENQC is a new method in studying quantum communication based on entanglement in quantum networks.

### Approximation of ENQC using effective radius

ENQC can be analyzed approximately using the physical meanings of *r*_*at*_ and *r*_*ap*_. Suppose Alice would like to transmit qubits to Bob, two situations can be considered: (i) Point-to-point quantum communication. In this case Alice and Bob both can access only one node in the network; (ii) Multipartite-to-multipartite quantum communication. We suppose that in (ii) the sender and the receiver each possess *k* nodes in the network and each node is independent from other nodes. That is, the distance of any two of the *k* nodes possessed by Alice (Bob) is larger than 2*r*_*ap*_. For convenience we further suppose that in (i) and (ii) Alice’s group of nodes (one or more) are far away from Bob’s group of nodes (one or more). Since the distance *d* in the expression of ENQC is supposed to be large here, all ENQC we mentioned here are *E*_0_ henceforth. Within this situation, our approach can approximate *E*_0_ in Eq.(2) of the main text.

We still consider the case of square lattice firstly. For the first situation (i), when *p* > 0.6, which is far beyond the percolation threshold and approximately corresponds to 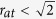, the effective circle contains only 5 nodes, 4 internal bonds and 12 peripheral bonds connected to the outside of the circle. When Alice and Bob are far away from each other, the quantum channels between Alice and Bob depend mostly on number of maximally entangled states induced by those 4 internal bonds for Alice or Bob. Only pairs of internal bonds belonging respectively to two sides can possibly form independent quantum channels. The number of established pairs is denoted by *X*_*p*_, which is a random variable. The expectation of the number of pairs is,


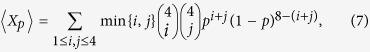


where 
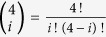
. We remark that when the number of participating nodes is large, min{*i*,*j*} can be omitted. The above equation equals to 4*p*. When *p* → 1, the medium can be seen as fully connected, and we have an asymptotic limit, 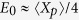.

For general *p* > 0.6, we notice that for a connected neighboring point of Alice, the probability that at least one peripheral bond is connected to the outside is (1 − (1 − *p*)^3^). Similar consideration can be applied for farther steps from Alice. Finally, the probability that the quantum connection does not meet a dead end in the medium can be written as (1 − (1 − *p*)^3^)^*α*^, where *α* is like an index of the medium depending only on lattice which can be fitted by numerical data. In this way, an estimated ENQC can be written as follows,


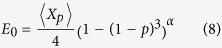


We can find that this formula agrees well with the data, see Fig. 4 in the main text.

As for a single realization, it is important to calculate the variance of *X*_*p*_ as well. Since the probability for having *m* quantum channels is


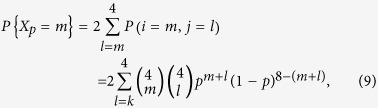


we arrive at the variance


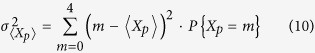


Next, we consider situation (ii). In this situation Alice can access *k* independent points *A*_1_, *A*_2_,…, *A*_*k*_, and Bob can similarly access *B*_1_, *B*_2_,…, *B*_*k*_. The same reasoning as in (i) can be applied. Eq. (7) and Eq. (8) can be generalized as


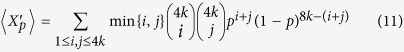


and


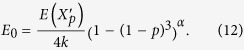


We note that here we assume *p* is far beyond the percolation threshold, as we already mentioned. On the one hand, higher order of correction terms become significant and thus not negligible when the considered *p* gets closer to the critical point. Actually the number of correction terms tend to infinity, when the estimation is carried out at the critical point of the network. On the other hand, the attractive radius becomes large when *p* close to the percolation threshold. Thus this approach would be complicated near the percolation threshold.

Our discussion here can be easily extended to general situations where nodes in a graph have degree *Z*, simply by changing Eq. (7) into


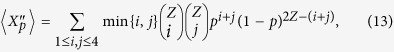


The variance of 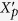 and 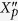 can be calculated as that of *X*_*p*_.

### Direct counting of ENQC by enumeration

**Figure 6:**
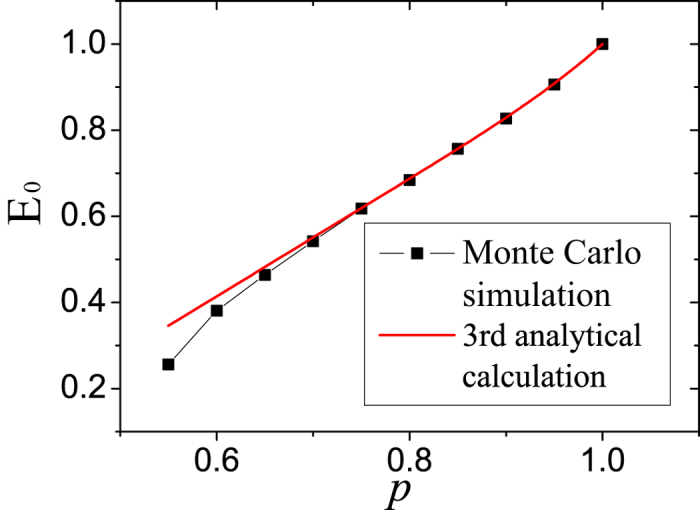
ENQC (*E*_0_) approximated up to third-order compared with Monte Carlo simulation. These two results agree with each other. Thus the method of calculating ENQC using effective radius can be regarded as a more succinct approximation.

We know that *E*_0_ is the asymptotic value of ENQC. Alternatively, we may not go directly by introducing the index *α* to approximate *E*_0_, as introduced in the main text and above. Here we try to write explicit *E*_0_ up to the third-order, which means third step from both the sender and the receiver, by purely enumerating different situations. This quantity should be already very close to the long-distance ENQC when *p* is rather great thus higher-order terms can be omitted. We have the following results,


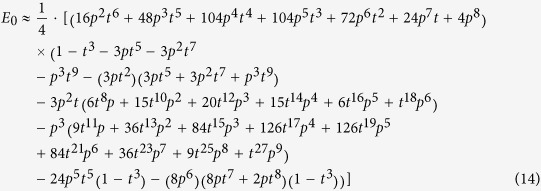


where *t* = 1 − *p*. In this equation we take into account the probability of a certain bond meeting an end up to 3 steps away from the original node and the probability of bonds which obstruct each other. In Fig. 6 we can find that this quantity already agrees well with Monte Carlo simulations when *p* > 0.7. By those observations, Eq. (5) in the main text is expected to be concise while being a good approximation.

## Additional Information

**How to cite this article**: Chen, X. *et al.* Expected number of quantum channels in quantum networks. *Sci. Rep.* **5**, 12128; doi: 10.1038/srep12128 (2015).

## Supplementary Material

Supplementary Information
