# Supplementary material for: Expected number of quantum channels in quantum networks
Source: Sci Rep. 2015 Jul 15;5:12128. doi: 10.1038/srep12128 (PMC4502510; doi:10.1038/srep12128)
Supplement: Supplementary Information [file srep12128-s1.pdf]

Supplementary Information: Expected number of quantum channels in quantum networks

Xi Chen,[1] He-Ming Wang,[1] Dan-Tong Ji,[2] Liang-Zhu Mu,[1,*] and Heng Fan[3,4,†]

[1] *School of Physics, Peking University, Beijing 100871, China*

[2] *Department of Physics, Tsinghua University, Beijing 100084, China*

[3] *Institute of Physics, Chinese Academy of Sciences, Beijing 100190, China*

[4] *Collaborative Innovation Center of Quantum Matter, Beijing 100190, China*

(Dated: May 17, 2015)

* muliangzhu@pku.edu.cn

† hfan@iphy.ac.cn

I. MORE ABOUT EFFECTIVE RADIUS

A. Explicit curves of ENQC with respect of distance

According to Eq. (2) and Eq. (4) in the main text, E_0 and r_{at} (r_{rp}) are two important parameters of a certain network. They represent the asymptotic values and the exponential-decaying behavior of the efficiency of quantum information transmission in quantum networks. Here, we show ENQC of different situations individually. All of them can serves as examples that different ENQC converge quickly to a fixed value as the distance increases. The results given by Monte Carlo simulation and the formulas are presented in FIG. S1, in which we can see that Eq. (2) and Eq. (4) agrees well with the numerical results.

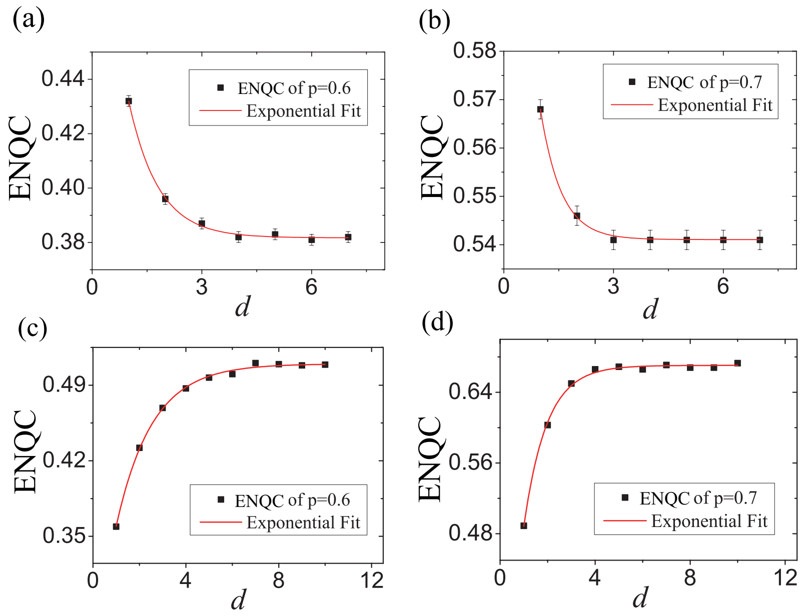

FIG. S1. The exponential convergence of ENQC. **(a)** $p = 0.6$, the attractive radius $r_{at} \approx 1.33$, and the asymptotic value of ENQC for long distance $E_0 \approx 0.38$. **(b)** $p = 0.7$, the attractive radius $r_{at} \approx 1.09$, and the asymptotic value of ENQC for long distance limit $E_0 \approx 0.54$. **(c)** $p = 0.6$, the repulsive radius $r_{rp} \approx 2.06$, and the asymptotic value of ENQC for long distance $E_0' \approx 0.5$. **(d)** $p = 0.7$, the repulsive radius $r_{at} \approx 1.46$, and the limit of ENQC for long distance is, $E_0' \approx 0.66$.

B. The attractive radius of other kinds of lattices and the universality about non-decaying medium

Here in FIG. S2 we provide three other examples of regular lattices in which attractive radii (circles) can be defined on the basis of Eq. (2) of the main text. The two lattices studied here are hexagon lattice and triangle lattice. The results are in support of that the effective radius of ENQC is expected as a universal phenomenon and the magnitude of ENQC can describe well the efficiency of quantum communication of various quantum networks.

We would like to explain the reason why the exterior of those effective circles behaves as an "non-decaying medium". Here we try to present a qualitative answer to this question by simply counting the number of outgoing edges (bonds) at each step begin from the source, for example, node occupied by Alice. Taking square lattice as a concrete example, the average number of edges connected to a certain node is $4p$. This quantity minus the edge which leads us to this node (input), we would have $4p-1 > 1$ as long as $p > 0.5$. So on

average the number of outgoing bonds at each step will increase exponentially so that it would be less possible for all choice an outgoing path to meet ends far away from the starting point. Roughly speaking, when the distance from the source is increasing, the effect of one particular disconnected bond is negligible among large number of outgoing bonds. That is why the exterior of the circles could be treated as an "non-decaying medium". Similar explanation could apply to other kinds of lattices. While for 1D quantum network, it is known that the efficiency of quantum communication is exponential decaying, which can be explained that the number of available bonds does not increase exponentially with the distance in compensation.

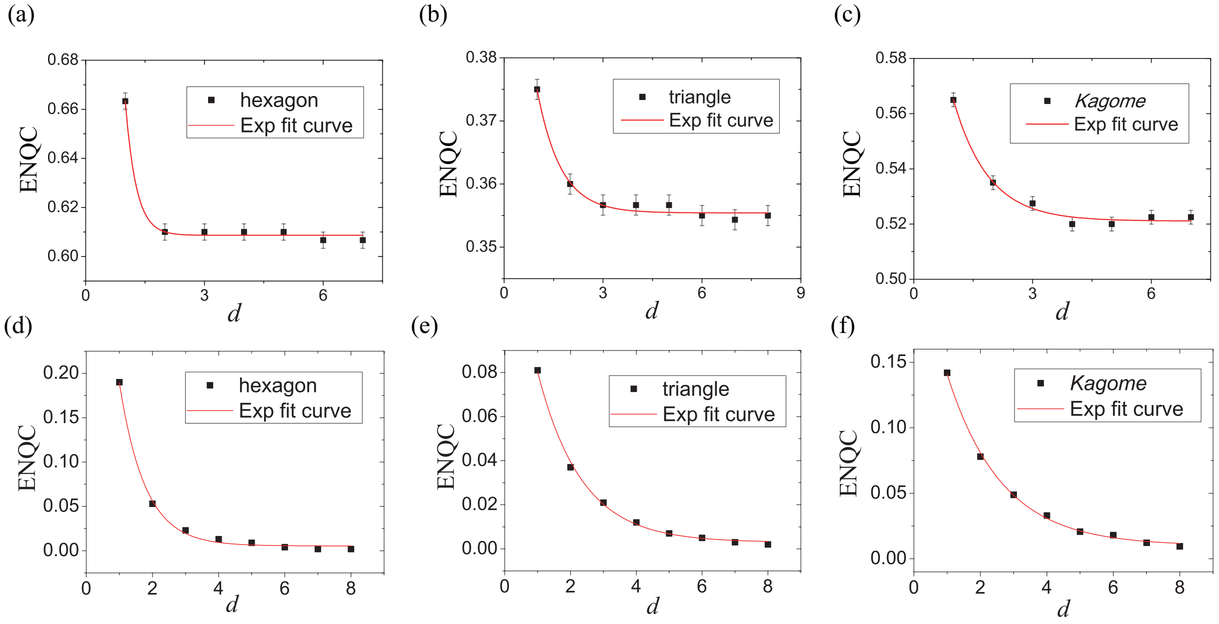

FIG. S2. **(a)** ENQC of hexagonal lattice with $p = 0.8$ and corresponding attractive radius $r_{at} = 1$, the lower-bound of r_{at}. **(b)** ENQC of triangle lattice with $p = 0.5$ and $r_{at} = 1.21$. **(c)** ENQC of *Kagomé* lattice with $p = 0.7$, $r_{at} = 1.38$. **(a)**, **(b)** and **(c)** are examples of $p > p_{th}$, which corresponds to the circumstances where the long-distance ENQC $E_0 > 0$, i.e., the ENQC would not decay to zero with greater distance. **(d)** ENQC of hexagonal lattice with $p = 0.5$ and corresponding attractive radius $r_{at} \approx 1.27$. **(e)** ENQC of triangle lattice with $p = 0.25$ and $r_{at} \approx 1.84$. **(f)** ENQC of *Kagomé* lattice with $p = 0.4$, $r_{at} \approx 2.14$. **(d)**, **(e)** and **(f)** are examples of $p < p_{th}$ respectively, which corresponds to $E_0 = 0$.

To give a qualitative view about the role of structure, we present a transverse comparison among these three kinds of network with same $p = 0.8$ (Fig. S3). The node degree of hexagon, *Kagomé* and triangle networks are 3, 4 and 6, respectively. We could see that, without quantum operation, for example, the entanglement swapping described in the main text, the ENQC tends to increase with larger node-degree and has a slower decrease with distance. In a qualitative view, given the same p, a network-structure with larger node-degree tends to be better connected than other structures with smaller node-degree, thus a larger node-degree gives larger ENQC.

C. The relationship between effective radius and randomness

We have already observed that the ENQC decreases exponentially and converge to a fixed value quickly, which can be interpreted by attractive radius. This phenomenon appears in every regular lattices studied in this work, such as square lattice, honeycomb, triangle and *Kagomé*. We guess that the effective radii

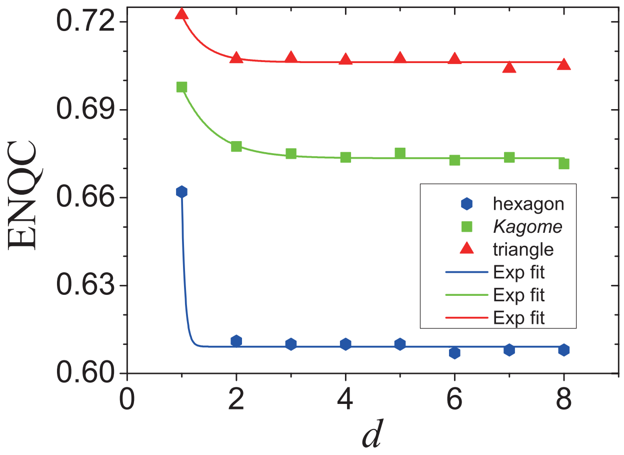

FIG. S3. Transverse comparison of ENQC among hexagon, *Kagomé* and triangle lattice. Symbols are simulation results and lines are exponential fitting curves. The node degree of hexagon, *Kagomé* and triangle networks are 3, 4 and 6, respectively. Among these networks, triangle networks have the largest E_0, the smallest difference between E_0 and $E(d=1)$, with the corresponding largest node-degree.

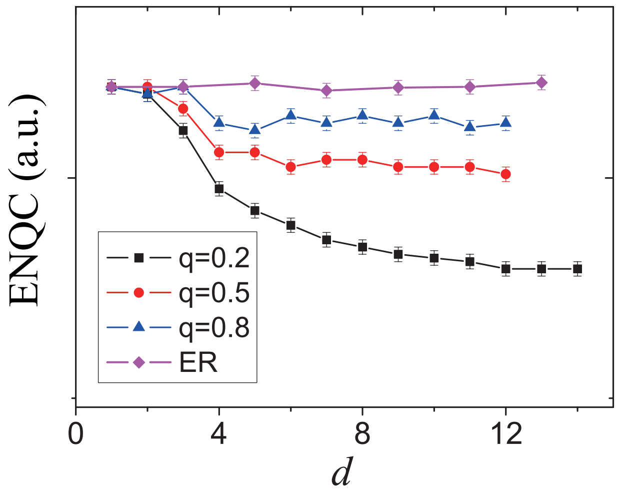

FIG. S4. Monte Carlo estimation of ENQC of SW networks with different switching probability q (black squares, red circles, and blue triangles represent the results of SW network with $q = 0.2$, 0.5, and 0.8, respectively. Lines are direct connection between symbols) and ENQC of an ER network(purple triangles). Because before the random switching of bonds, each node is connected to the 3 nearest nodes both clockwise and counterclockwise, we observed a slow decrease within $d \leq 3$. Within $d \geq 3$ we observe an exponential decay in the curve of $q = 0.2$. However, the exponential decay is less explicit for $q = 0.5$ and $q = 0.8$. The curve of ENQC for higher q is getting closer to the curve of an ER network, which is a completely random network. The expectation ENQC value of those purple triangles would be equal.

might arise from the short-distance regularities for those lattices. To support this conjecture, we investigate some random graphs. For example, a Small-World network[1] is a random network whose "randomness" can be controlled by a parameter, rewiring probability q. Two kinds of Small-World networks with extreme q are regular lattices (regular network) and the Erdős-Rényi (ER)[2] network, namely the completely random graph. We found that the effective radii phenomena disappears gradually when the "randomness" of the network increases. Following is our detailed investigation.

Erdős-Rényi network and its radius—Erdős-Rényi (ER) network is a completely random network [2]. An

ER-quantum network with N nodes can be operated as follows: (i) $N(N-1)/2$ non-maximally entangled pairs are shared between nodes, forming a complete graph. (ii) Each pair is converted into maximally entangled pair with singlet conversion probability p. Because all nodes of an ER network can be treated as identical, we predict that there will be no effective radius in ER network. When doing our Monte Carlo estimations we align the nodes of our ER network on a straight line so that we can define distance in this system. The purple squares in FIG. S4 represent numerical results. Those purple squares are almost located in a horizontal line, which indicates the ENQC of an ER network does not vary with distance, as expected.

Small-World network and its radius—Compared with Erdős-Rényi network, generally a Small World (SW) network possesses less randomness [1]. Here we consider the following version of SW-quantum network: First, N nodes are aligned as a circle with each point sharing entangled pairs with $\frac{K}{2}$ nearest points clockwise and $\frac{K}{2}$ nearest points counterclockwise. Second, every bond is switched to a new end with a probability q, like the transportation of entangled particles. That is, if the nodes are labeled $n_0 \dots n_{N-1}$, this rewiring process is done by replacing($i < j$) bond (n_i, n_j) with (n_i, n_k) where k is a randomly chosen node from all possible ones except (i) $k = i$ or $k = j$ (ii) $k = k'$ while there is already an bond (n_i, n'_k) existing. Third, each entangled pair is converted to maximally entangled state with probability p. In other words, its random switching of bonds (random entangled distribution) happens after a first determined bond distribution. The probability of switching bonds acts as a parameter of randomness. The three other curves in FIG. S4 correspond to SW network with switching probability $q = 0.2, 0.5$, and 0.8, respectively. Here we have fixed singlet conversion probability $p = 0.5$, and $K = 6$. As q increases, the randomness of the network grows higher and both the graph configuration and the long-distance behavior of ENQC is approaching that of an ER network. Note that a SW network with $q = 1$ is just an ER network since it is completely random. The configuration of our SW network is presented in FIG. 3(a) of the Main Text.

As for the radius, we can firstly notice that there is a slow decrease of ENQC when $d < 3$. This is because our Monte Carlo estimation we set $K/2 = 3$, that is, each node is connected with the nearest 3 nodes both clockwise and counterclockwise. These connections are partly remained after the switching process such that a certain node is better connected to nodes with $d \leq 3$ than others. So we investigate possible exponential decay of ENQC in the range $d \geq 3$. Note that for $q = 0.2$, only a small part of bonds is switched and the total network remains not very far from 'regular'. There is a clear exponential decay of ENQC such that we could well define the effective radius. This fact is what we have presented for several regular lattices. Then for $q = 0.5$ and 0.8, the network changes away from 'regular' network. The numerical curves do not explicitly show exponential decay so the effective radii do not exist in such cases.

To summarize, we propose that the attractive and repulsive radius, i.e. an exponential form of the ENQC-d function, is a featured short-distance structure of regular lattices for quantum network. The more regular the structure is, the better such radius can be defined. Thus we consider that the reason that ENQC converge quickly to fixed values may originate from the short-distance bonds structure of the regular lattices.

II. LATTICE TRANSFORMATION FROM *KAGOMÉ* LATTICE TO SQUARE LATTICE

Via entanglement swapping processes on certain nodes of a *Kagomé* lattice, the lattice can be transformed into a square lattice[3]. According to Ref. [4], the percolation threshold, p_c, of a *Kagomé* lattice is slightly higher than that of a square lattice, which is p_c(square)=0.5. As we know before, entanglement swapping could probably change a *Kagomé* lattice under threshold to a square lattice above threshold. Now by our analyzing, it can be obtained from the numerical curves that entanglement swapping enhances the ENQC (E_0) for all $p > 0.55$ (actually it holds for all $p > 0.5$ because the ENQC (E_0) of *Kagomé* lattice, with a higher percolation threshold, decreases more quickly than the ENQC (E_0) of square lattice near threshold.). This result is presented in FIG. S5.

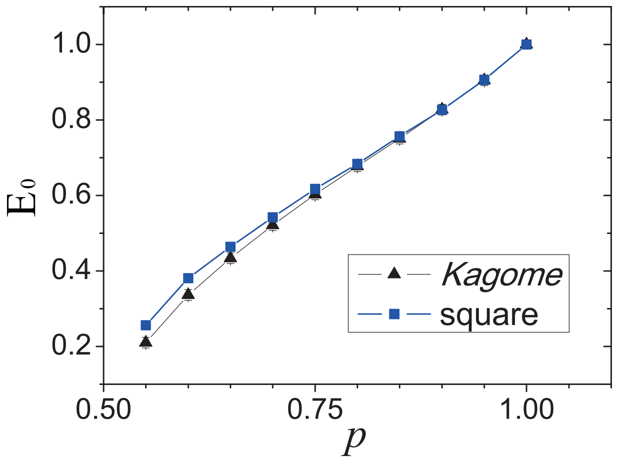

FIG. S5. Monte Carlo estimation of ENQC (E_0) before and after entanglement swapping, from *Kagomé* lattice to square lattice.

III. ENQC IN RANDOM NETWORKS WITH ARBITRARY DEGREE-DISTRIBUTION

Here we would discuss the application of our notion in random networks with arbitrary degree-distribution, for example, quantum complex networks[5]. Technically, a random network is defined by a generating function of node degree-distribution[6]

$$G_0(x) = \sum_{k=0}^{\infty} q_k x^k \tag{1}$$

where $G_0(x)$ is the generating function, $\{q_k\}$ is the node degree-distribution. q_k gives the probability of a node with degree k. The network configuration is determined probabilistically. However, this degree distribution is universal for every nodes such that either in the sense of averaging over all nodes, or averaging over a network ensemble, all nodes in the network can be treated equally. The ENQC only varies with distance and singlet-conversion probability p, not their absolute position. Thus, as long as a distance can be defined in a random network, our notion can also be applicable.

The cost of network can also be determined, we can calculate the mean degree of nodes in the network

$$\langle k \rangle = G_0'(1), \tag{2}$$

which represents the cost of the network and corresponds to N_0 in Eq. (1) of the main text. Thus we can define ENQC of a quantum complex-network in the same way. And the notion of effective radius and the calculation of E_0 can be determined similarly.

IV. THE COMMUNICATION COMPLEXITY FROM A SENDER AT A GIVEN NODE

Now we choose a certain point A as our starting point (sender) and study the communication between this node A and other nodes in the network. It is obvious that the result ENQC would depend on the degree of the receiver B, the distance between A and B, i. e., the position of B, and the singlet-conversion probability.

Following the idea of calculating E_0 presented in the Methods section, we can similarly derives the number of matches $\langle X(k_A, k, p)\rangle$ and $E_0(k_A, k, p)$ after first-order cutoff approximation

$$\langle X(k_A, k, p)\rangle = \sum_{1\le i\le k_A, 1\le j\le k} \min\{i, j\} \binom{k_A}{i} \binom{k}{j} p^{i+j}(1-p)^{k_A+k-(i+j)}, \tag{3}$$

$$E_0(k_A, k, p) = \frac{\langle X(k_A, k, p)\rangle}{N_0}(1-(1-p)^{N_0-1}), \tag{4}$$

where k_A is the degree of node A, k is the degree of node B, p is the singlet conversion probability. Also, N_0 represents the cost of network, which can be replaced by $\langle k\rangle$, for a complex network.

We could see that E_0 under this circumstances, acts like a pair-correlation function. Note that this correlation tends to be saturated for $k_A \gg k$ or $k_A \ll k$. For example, **(i)** $k_A \gg k$, we have $i \gg j$, $\min\{i,j\} = j$, $\langle X(k_A, k, p)\rangle = pk$,

$$E_0(k_A, k, p) = E_0(k, p) = \frac{p(1-(1-p)^{N_0-1})}{N_0}k \leq \frac{pk}{N_0}; \tag{5}$$

(ii) $k_A \ll k$, we have $i \ll j$, $\min\{i,j\} = i$, $\langle X(k_A, k, p)\rangle = pk_A$,

$$E_0(k_A, k, p) = E_0(k_A, p) = \frac{p(1-(1-p)^{N_0-1})}{N_0}k_A \leq \frac{pk_A}{N_0} \tag{6}$$

This interesting result indicates that the E_0 is saturated if the node with smaller degree is saturated, namely E_0 is bounded by the smaller degree of the degree of the sender and the receiver. Given the same N_0 and p, a network with a small range of node-degree distribution have higher communication efficiency than a network with a large range of degree distribution.

---

[1] Watts, D. J. & Strogatz, S. H. Collective dynamics of 'small-world' networks. *Nature* (London) **393**, 440 (1998).

[2] Erdős, P. & Rényi, A. On random graphs I. *Publicationes Mathematicae* **6** 290 (1959).

[3] Lapeyre, G. J., Wehr, J. & Lewenstein, M. Enhancement of entanglement percolation in quantum networks via lattice transformations. *Phys. Rev. A* **79**, 042324 (2009).

[4] Grimmett, G. *Percolation 2nd edn.* (Springer-Verlag, Berlin, 1999).

[5] Cuquet, M. & Calsamiglia, J. Entanglement Percolation in Quantum Complex Networks. *Phys. Rev. Lett.* **103**, 240503 (2009).

[6] Newman, M. E. J., Strogatz, S. H. & Watts, D. J. Random graphs with arbitrary degree distributions and their applications. *Phys. Rev. E* **64**, 026118 (2001).
